# Genome instability and pressure on non-homologous end joining drives chemotherapy resistance via a DNA repair crisis switch in triple negative breast cancer

**DOI:** 10.1093/narcan/zcab022

**Published:** 2021-06-15

**Authors:** Adrian P Wiegmans, Ambber Ward, Ekaterina Ivanova, Pascal H G Duijf, Mark N Adams, Idris Mohd Najib, Romy Van Oosterhout, Martin C Sadowski, Greg Kelly, Scott W Morrical, Ken O’Byrne, Jason S Lee, Derek J Richard

**Affiliations:** Queensland University of Technology (QUT), Cancer and Ageing Research Program, Centre for Genomics and Personalised Health, School of Biomedical Sciences, Translational Research Institute, Woolloongabba QLD 4121, Australia; School of Medicine, University of Queensland, Herston, QLD, 4006, Australia; School of Medicine, University of Queensland, Herston, QLD, 4006, Australia; Epigenetics and Diseases Laboratory, QIMR Berghofer Medical Research Institute, Herston, QLD, 4006, Australia; Queensland University of Technology (QUT), Cancer and Ageing Research Program, Centre for Genomics and Personalised Health, School of Biomedical Sciences, Translational Research Institute, Woolloongabba QLD 4121, Australia; Queensland University of Technology (QUT), Cancer and Ageing Research Program, Centre for Genomics and Personalised Health, School of Biomedical Sciences, Translational Research Institute, Woolloongabba QLD 4121, Australia; Centre for Data Science, Queensland University of Technology (QUT), Brisbane, QLD, Australia; University of Queensland Diamantina Institute, University of Queensland, Brisbane, QLD, Australia; Queensland University of Technology (QUT), Cancer and Ageing Research Program, Centre for Genomics and Personalised Health, School of Biomedical Sciences, Translational Research Institute, Woolloongabba QLD 4121, Australia; Queensland University of Technology (QUT), Cancer and Ageing Research Program, Centre for Genomics and Personalised Health, School of Biomedical Sciences, Translational Research Institute, Woolloongabba QLD 4121, Australia; Epigenetics and Diseases Laboratory, QIMR Berghofer Medical Research Institute, Herston, QLD, 4006, Australia; Queensland University of Technology (QUT), Cancer and Ageing Research Program, Centre for Genomics and Personalised Health, School of Biomedical Sciences, Translational Research Institute, Woolloongabba QLD 4121, Australia; School of Medicine, University of Queensland, Herston, QLD, 4006, Australia; Epigenetics and Diseases Laboratory, QIMR Berghofer Medical Research Institute, Herston, QLD, 4006, Australia; Department of Biochemistry, Larner College of Medicine, University of Vermont, Burlington, VT 05405, USA; Queensland University of Technology (QUT), Cancer and Ageing Research Program, Centre for Genomics and Personalised Health, School of Biomedical Sciences, Translational Research Institute, Woolloongabba QLD 4121, Australia; School of Medicine, University of Queensland, Herston, QLD, 4006, Australia; Epigenetics and Diseases Laboratory, QIMR Berghofer Medical Research Institute, Herston, QLD, 4006, Australia; School of Biomedical Sciences, Queensland University of Technology, Brisbane, QLD, Australia; Queensland University of Technology (QUT), Cancer and Ageing Research Program, Centre for Genomics and Personalised Health, School of Biomedical Sciences, Translational Research Institute, Woolloongabba QLD 4121, Australia

## Abstract

Chemotherapy is used as a standard-of-care against cancers that display high levels of inherent genome instability. Chemotherapy induces DNA damage and intensifies pressure on the DNA repair pathways that can lead to deregulation. There is an urgent clinical need to be able to track the emergence of DNA repair driven chemotherapy resistance and tailor patient staging appropriately. There have been numerous studies into chemoresistance but to date no study has elucidated in detail the roles of the key DNA repair components in resistance associated with the frontline clinical combination of anthracyclines and taxanes together. In this study, we hypothesized that the emergence of chemotherapy resistance in triple negative breast cancer was driven by changes in functional signaling in the DNA repair pathways. We identified that consistent pressure on the non-homologous end joining pathway in the presence of genome instability causes failure of the key kinase DNA-PK, loss of p53 and compensation by p73. In-turn a switch to reliance on the homologous recombination pathway and RAD51 recombinase occurred to repair residual double strand DNA breaks. Further we demonstrate that RAD51 is an actionable target for resensitization to chemotherapy in resistant cells with a matched gene expression profile of resistance highlighted by homologous recombination in clinical samples.

## INTRODUCTION

The clinical use of chemotherapy for hard-to-treat cancers has meant prospective continued emergence of chemotherapy resistance that drives poor patient outcome (1). Invariably during relapse, the secondary cancers are resistant to the therapy initially utilized (2). For our studies, we exploited the triple negative breast cancer subtype to model chemotherapy resistance. Triple negative breast cancer (TNBC) is characterized by the absence of the therapeutically targetable hormone receptors namely estrogen, progesterone and human epidermal growth factor 2 receptors. TNBC is fast-growing and elicits pressure on the DNA repair pathways, even in the absence of therapy, via endogenous replication stress (3). Consequently, chemotherapy-induced DNA repair functions can become dysregulated via hyper-repair or deficiency as a means to stabilize the cancer genome resulting in chemoresistance. We sought out to define the molecular mechanisms of DNA repair driving chemoresistance.

The major DNA double strand break repair pathways available to TNBC cells are the low fidelity, non-homologous-end joining pathway mediated by the kinase DNA-PK and the high fidelity homologous recombination repair pathway mediated by the recombinase RAD51 (4). The activation of the DNA damage response (DDR) is a result of cell cycle stage and availability of appropriate machinery in either of the pathways. There are common genotypic trends in TNBC that guide the DDR. Of note, virtually all (>80%) TNBCs harbor p53 mutations (5,6), 73% display RAD51 overexpression and 15% have BRCA1/2 mutations (7). In TNBC, overexpression of p53 promotes non-homologous end joining throughout the cell cycle by repressing RAD51 gene transcription and activating 53BP1 (8), supporting non-homologous end joining. In contrast homologous recombination deficiency (HRD) can be overcome in the BRCA1^mutant^ subset via RAD51 overexpression compensation for BRCA1 loss (9). This is one of the molecular mechanisms observed for the low objective response rates of BRCA-mutant patients to PARP inhibitors (9). A variety of BRCA1-proficient tumour types also display high levels of nuclear RAD51 without DNA damage induction (10,11), which suggests a role for RAD51 overexpression in tumorigenesis and possibly chemotherapy resistance (7,9). We have previously shown that TNBC metastasis require high RAD51 expression and homologous recombination (7). We speculate that chemoresistant-driven metastasis may also require high RAD51 expression and homologous recombination, even though repair mechanisms in response to chemotherapy is dominated by non-homologous end joining (12,13).

Currently indirect measurement of DNA repair function is in clinical use as a diagnostic for TNBC. HRD score is the arithmetic sum of chromosome instability based on somatic sequencing. These mutational signatures differ due to patient variation in aberrant DNA repair function (14). Clinically, HRD score is an important metric in providing a ‘snapshot’ for aberrant DNA repair, *BRCA*ness profile and chemoresistance (15). While HRD score has found utility to identify patients likely to respond to PARP inhibitors and define cellular response to frontline chemotherapy in TNBC (17), there were no differences in objective response rates in patients with high or low HRD for chemotherapy that induces DNA damage, namely docetaxel and carboplatin in unselected patients (16). Therefore, genomic analysis alone falls short in predicting cellular and/or patient response and the mechanisms have not been fully defined. We hypothesize that functional DDR mechanisms, expression and signaling may represent clinically relevant prognostic readouts beyond mutational scoring to guide clinical therapeutic choices. In support of this hypothesis a functional assay for homologous recombination (RAD51 foci count) outperformed HRD score in prognostic evaluation of patient response to PARP inhibition (18). An in-depth investigation into functional DNA pathway response in the chemoresistance setting is required and has yet to be done. We aim to elucidate and exploit key molecular drivers as complementary rational therapeutic targets for the more optimal treatment of TNBC.

## MATERIALS AND METHODS

### Gene expression signature

Changes in gene expression using Affymetrix Human Genome arrays between patients treated with a combination of docetaxel and doxorubicin, comparing TNBC to the Luminal and HER2+ subtypes (GSE25066 (19)), identified 88 and 130 genes that were significantly up- or downregulated, respectively (Supplementary Table 1). A weighted gene expression signature score was determined from these data. If individual genes were represented by multiple probes, probe-level folds change were collapsed to gene-level fold change by averaging the folds change for each probe. For each of the genes, level 3 mRNA levels from The Cancer Genome Atlas (TCGA) HiSeq RNAseq V2 breast cancer dataset were used to determine expression level *z* scores on a per-gene basis. For each sample, *z* scores for all of the genes contained in the signature were multiplied by the respective up- or down-regulation factors shown in Supplementary Table 1, generating a final weighted gene expression signature score for each sample. Breast cancer subtypes were defined according to the PAM50 classifier.

### Cell culture

MDA-MB-231, MDA-MB-468, Hs578t and BT549 cells were sourced from the ATCC. Cells were confirmed to be negative for mycoplasma and authenticated by short tandem repeat analysis every 6 months. All lines were maintained in Dulbecco's Modified Eagle Medium with 5% fetal bovine serum (FBS) and antibiotics, except BT549, which was maintained in RPMI with 5% FBS and antibiotics.

### Chemotherapy adaption

Cell lines above were grown in the presence of ever-increasing doses of doxorubicin and docetaxel over the course of 6–8 months. Cellular death above 80% meant cells were allowed to recover for 1–2 weeks in media free of the chemotherapy combination.

### Chemotherapy dose-response analysis, targeted treatment and readout

Cells were seeded in 24-well plates (5 × 10^4^/well), then incubated for 72 h in the presence of docetaxel or doxorubicin (0–100 μM), the combination of 1:100 docetaxel or doxorubicin referred to as ‘chemotherapy or CHEMO’ (0.3 and 30 nM), small molecules targeting DNA-PK (0–1 mM). Metabolic activity was assayed A 5 mg/ml stock solution was prepared with Thiazolyl Blue Tetrazolium Blue (MTT) and DPBS and read at 590 nm after 60 min incubation and dehydration with 10% total volume isopropanol.

### Incucyte Zoom realtime growth analysis

Cell proliferation and/or cell death over time was evaluated by live cell imaging using the IncuCyte Zoom (Essen BioScience). For cell death assessment, Nuc Green Live dead reagent was added to culture medium (1:200) when treatment and vehicle control conditions were added. The Phase channel was used to measure cell proliferation/total cells (measured as % phase confluence) and the Green channel was used to detect cell death (measured as % green confluence). Plates were scanned at 3 hourly intervals to capture images. Data from IncuCyte Zoom was exported into Graphpad Prism to calculate the Area Under the Curve for Phase Confluence and Green Confluence.

### Non-homologous end joining and Homologous recombination assay

In a six-well plate, adherent cells stably expressing pDR-GFP (for HR) were transiently transfected using Lipofectamine 2000 with 2 μg of linearized GFP reporter vector (for NHEJ) or 2 μg pCBASceI (Clontech) to cleave GFP (for HR) and circular GFP to serve as a separate control for transfection efficiency in each assay. GFP expression was measured on the FACS-Canto II flow cytometer (BD Biosciences) 72 h later. Due to the differences in transfection efficiency between individual cell lines, each assay was normalized for control circular GFP.

### Immunofluorescence

In short, 5 × 10^4^ cells were seeded onto 18 mm glass coverslips, gamma-irradiated with 2 Gy then time points up to 24 h later, washed in PBS, fixed with cold 4% paraformaldehyde and permeabilized with 0.1% Triton X-100 for 15 min at room temperature (RT). Cells were blocked with 1% BSA, incubated with primary antibody (1:50 in 0.1% BSA + PBS, anti-RAD51, anti-53BP1 and anti-phospho-DNAPK S2056) overnight at 4°C then secondary antibody (1:500 Abcam, anti-mouse Cat#ab150113, anti-rabbit Cat#ab6564) for 30min at RT. Immuno-stained cells were then washed, mounted with ProLong^®^ Gold Antifade Mountant containing 4′,6′-diamidino-2-phenylindole (DAPI) for microscopy. Images were taken on a Zeiss 780-NLO confocal microscope.

### Statistical analysis

Results are presented as mean ± SEM of replicate analyses and are either representative or inclusive of at least three independent experiments. In all Figures, significant differences between specified pair of conditions were assessed using two-tailed Student's *t*-tests, with *P* values *<0.01, **<0.001, ***<0.0001, ****<0.00001 considered significant. Inhibitory concentration 50% (IC50) values were calculated by interpolation of sigmoidal dose-response curves produced from non-linear regression analyses.

## RESULTS

### Chemoresistance to the combination of doxorubicin and docetaxel is efficiently achieved by loss of genetic material

**Figure 1.**
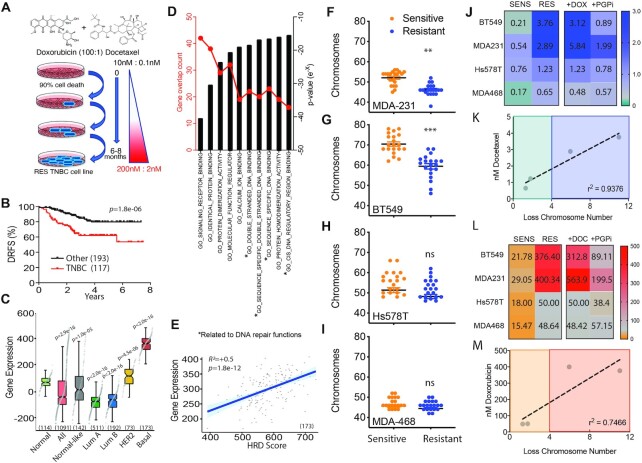
Evaluation of TNBC genome stability traits from clinical trials and drug adapted cell lines. (**A**) Schematic flow diagram of method t establish the chemoresistant cell lines. (**B**) Distance relapse free survival of neoadjuvant doxorubicin and docetaxel treated patients stratified for triple negative breast cancer subtype versus the remaining subtypes (GSE25066; 310 patients in total). *P* = 1.8e-6 Log-rank Mantel-Cox test. (**C**) In an independent dataset, the breast cancer dataset from The Cancer Genome Atlas (TCGA), the molecular signature derived from GSE25066 (see Methods and Supplementary Table 1) perfectly separates all 173 basal breast cancer samples from all 703 luminal samples. Subtypes were defined according to the PAM50 classifier. Mann-Whitney *U* test p-values as shown. (**D**) Molecular signatures Database curated gene set comparing TNBC to other subtypes for gene expression changes in molecular functions ranked based on gene numbers and statistical significance following neoadjuvant doxorubicin and docetaxel chemotherapy (GSE25066). Mann–Whitney *U* test *P*-values as shown. (**E**) Patient samples compared for genes expression induced in response to chemotherapy combination doxorubicin and docetaxel profile compared to Homologous recombination deficiency measured using a gene expression signature, based on the expression of 230 genes (HRD_score), as previously reported (46). (**F**) Chromosome count of metaphase spreads comparing MDA-M-231 sensitive and resistant cell lines. (**G**) BT549, (**H**) Hs578t and (**I**) MDA-MB-468 (***P < 0.0001, **P < 0.001 paired Student *t*-test, ± SEM of three independent experiments). (**J**) Heat map of IC50 values derived from dose curves for each matched sensitive (SENS) and drug adapted (RES) TNBC cell lines for docetaxel monotherapy, in combination with doxorubicin and with the addition of P-glycoprotein pump inhibitor (PGPi). IC50 (nM) indicated from three independent experiments. (**J**) Correlation between IC_50_ of docetaxel and the average loss of chromosome number observed in the matched resistant cell line. *r^2^ =* 0.9376 Pearson correlation coefficient, *P = 0.034 paired student t-test. (**K**) As above in (I), heat map utilizing doxorubicin monotherapy in combination with docetaxel and with the addition of P-glycoprotein pump inhibitor (PGPi). (**L**) Correlation between IC50 of doxorubicin and the average loss of chromosome number observed in the matched resistant cell line. *r^2^ =* 0.7466 Pearson correlation coefficient, *ns*.

There is a clinical need to predict response following chemotherapy treatment and avoid unwarranted treatment and a cancer relapse. We created chemoresistant TNBC lines by escalating doses of the combination of docetaxel and doxorubicin in a ratio of 100:1 to mimic the clinical use (Figure 1A). We analysed the clinical survival data for standard of care neoadjuvant taxane-anthracycline (docetaxel and doxorubicin) frontline combination therapy comparing TNBC with Luminal/HER2+ subtypes. Results showed that TNBC patients displayed significantly worse distant recurrence free survival (GSE25066, ****P = 1.8^e-6^) (Figure 1B). To evaluate the essential pathways observed in TNBC patients in response to neoadjuvant docetaxel and doxorubicin frontline combination, we analyzed the gene expression changes in treated patients, comparing TNBC to the Luminal/HER2+ subtypes (GSE25066-Supplementary Figure S1) (19). Molecular Signatures Database curated gene set analysis resulted in differential induction of 88 genes, while 130 genes were repressed (Supplementary Table 1). These genes were able to constitute a molecular signature that perfectly separated out 173 basal breast cancer samples from a cohort of 1091 breast cancer patient samples (Figure 1C). Therefore, TNBC gene expression in response to chemotherapy was unique to that subtype. Gene ontology molecular functions analysis of the TNBC-distinguishing gene signature revealed four of the top 10 ranked, contained pathways with known DNA binding functions. Of these four, two included pathways with double strand DNA binding, which is functionally dominated by DNA repair proteins and associated functions (Figure 1D). Although chemoresistance has been attributed to cancer stem-cells, no gene ontology annotated terms or molecular functions were assigned to stem-like functions or phenotypes. This may suggest a dominant role for DNA repair pathways in gene expression response to docetaxel and doxorubicin. In line with this, we observe a positive correlation between our above gene expression signatures (induced and repressed genes) and a gene expression signature, based on the expression of 230 genes as measure for homologous DNA repair deficiency (HRD) in basal breast cancer patients (Figure 1E).

In order to evaluate the chemotherapy resistance mechanisms driving TNBC recurrence, we grew four TNBC cell lines MDA-MB-231, Bt549, Hs578t and MDA-MB-468 and exposed them to the frontline therapy combination of doxorubicin and docetaxel starting at 0.1 nM docetaxel and 10 nM doxorubicin and concluding with 2 nM docetaxel and 200 nM doxorubicin. The concentration was chosen to mimic the clinical administration of bolus doxorubicin and infusion of docetaxel (20). Resistance was achieved over 6–8 months with escalating doses of the chemotherapy combination. Chemotherapy resistance has been shown to correlate with acquisition of new genomic aberrations and adaptive copy-number evolution (17). To investigate this, we performed metaphase spread counts comparing chromosome numbers between sensitive and resistant paired lines. On a macromolecular level all resistant cell lines had an overall loss of genetic material compared to sensitive parental cells. There was significant loss in chromosome count in the MDA-MB-231 (Figure 1F) and BT549 (Figure 1G) and trended lower in Hs578t (Figure 1H) and MDA-MB-468 cells (Figure 1I) but did not reach statistical significance. The two statistically significant lines correlated with higher IC50 values achieved for docetaxel (Figure 1J) and there was a strong correlation between chromosome loss and resistance to docetaxel (Figure 1K). This observation was repeated with doxorubicin (Figure 1L/M). This suggests that adaptation to DNA damaging agents requires or results in loss of genetic material. A more detailed analysis of copy number variation in resistant MDA-MB-231 and MDA-MB-468 lines confirmed the observations made with our metaphase spread data (Supplementary Figure S2 and Supplementary Table S2). The more highly resistant MDA-MB-231 genetics displayed a greater increase in loss of heterozygosity and a reduction in copy number gains compared to the MDA-MB-468 resistant line (Supplementary Figure S2). More specifically MDA-MB-231 resistant shows copy number loss of p53, CDC25A, PUMA, BAX, MLH2, XPF, DNA-PK, 53BP1, RPA1 and BRCA1 not observed in MDA-MB-468 (Supplementary Table S2). In addition, there was a positive correlation between the TNBC-distinguishing gene signatures and several measures of genomic instability, including the CIN70 gene expression signature (functional aneuploidy), overall aneuploidy and chromosome arm aneuploidy burden (Supplementary Figure S3). Overall, we observed a correlation between chemotherapy-induced gene expression specifically in TNBC and DDR pathway activation/genome instability. We also observed a correlation between genome instability and levels of resistance achieved.

Each of the drugs doxorubicin and docetaxel are substrates for the multi-drug resistance efflux pump P-glycoprotein (Pgp) (21). We analysed the contribution of the pump to the overall resistance achieved in the resistant lines by the addition of the Pgp inhibitor tariquidar and observed that on average inhibition reduced the resistance IC50 by 2-fold across all lines, notably higher in the lines that achieves higher levels of resistance (Figure 1J/L).

### Chemoresistance drives a shift towards homologous recombination to sustain genome stability

**Figure 2.**
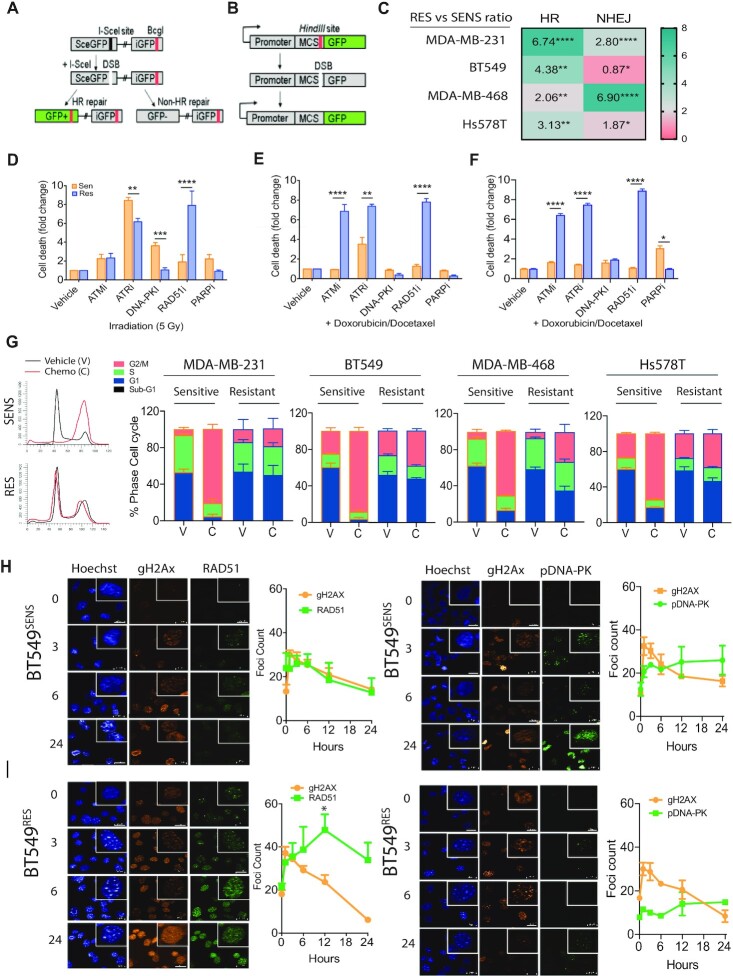
Functional shift in DNA damage response in drug adapted cell lines. (**A**) Schematic diagram of DNA repair GFP fluorescent reporter for homologous recombination driven repair of I-Sce1 enzyme-mediated double strand break. (**B**) Schematic diagram of DNA repair GFP fluorescent reporter for non-homologous end joining driven repair of linearized GFP. (**C**) All matched sensitive and resistant cell lines were assayed for homologous recombination activity and non-homologous end joining driven repair in non-synchronized cells. Fold change is expressed as a ratio comparing output of resistant cells compared to sensitive control **P < 0.001 paired Student t-test, ± SEM of three independent experiments. (**D**) MDA-MB-231 cells monitored for cell growth over 5 days with incucyte live cell imaging and dead cells selected for with a cell-impermeant dye. Response was compared for irradiation induced double strand breaks inhibition of key proteins against homologous recombination (RAD51), non-homologous end joining (DNA-PK), alternative non-homologous end joining (PARP) and key upstream signaling kinases (ATM and ATR) (***P < 0.0001 paired Student's *t*-test, ± SEM of 3 independent experiments). (**E**) Matched sensitive and resistant MDA-MB-231 cells lines were assayed as above, however double strand breaks were induced by the combined activity of low dose doxorubicin and docetaxel (****P < 0.00001, **P < 0.001 paired Student's *t*-test, ± SEM of three independent experiments). (**F**) Matched sensitive and resistant BT549 cells lines were assayed as above, however double strand breaks were induced by the combined activity of low dose doxorubicin and docetaxel (****P < 0.00001, **P < 0.001 paired Student's *t*-test, ± SEM of three independent experiments). (**G**) Cell cycle analysis by PI incorporation. Example trace of MDA-MB-231 matched sensitive and resistant, treated with chemotherapy (C) or DMSO, vehicle control (V). Stacked bar plots of cell cycle phase percentages ± SEM of three independent experiments. (**H**) Immunofluorescent analysis of the rate of double strand break repair marked by comparison of gammaH2AX foci marked breaks, homologous recombination RAD51 foci and phospho-DNA-PK (S2056) foci as a marker for switch to non-homologous end joining in BT549 sensitive cells. (**I**) Immunofluorescent analysis of the rate of double strand break repair marked by comparison of gammaH2AX foci marked breaks, homologous recombination RAD51 foci and phospho-DNA-PK (S2056) foci as a marker for switch to non-homologous end joining in BT549 resistant cells.

To evaluate the role of the individual DNA repair pathways in the observed chemoresistance profiles, we analysed the specific cellular double strand DNA break repair response. The two main double strand break DNA repair pathways are the error prone non-homologous end-joining pathway and high-fidelity homologous recombination pathway. We utilized the classical enzyme directed double strand DNA break of GFP and the MDA-MB-231 matched pair sensitive and resistant lines. The first assay was a measure of homologous recombination activity and required a sister template of GFP (iGFP) and active pathway to recapitulate the fluorescence of an enzyme cleaved GFP construct (Figure. 2A). The second assay was a measure of non-homologous end joining via recapitulation of the upstream gene cassette with active promoter for GFP expression (Figure 2B). In contrast to our expectations, related to genetic losses via high levels of error prone non-homologous end joining repair, the most highly resistant cell lines MDA-MB-231 and BT549 displayed significantly enhanced homologous recombination activity with 4–6-fold higher activity compared to the matched sensitive lines, while the lesser resistant lines Hs578t and MDA-MB-468 each displayed a moderate increase of 2–3-fold in homologous recombination activity (****P < 0.0001, ***P < 0.001, **P < 0.01, *P < 0.05, Figure 2C). Analysis of non-homologous end joining capacity revealed that MDA-MB-231 displayed moderate enhanced activity while BT549 displayed reduced activity. The lesser resistant line Hs578t had moderately enhanced activity at 1.8-fold, while MDA-MB-468 displayed greatly enhanced NHEJ activity (Figure 2C). MDA-MB-468^RES^ did not achieve high levels of resistance and seems to be more reliant upon non-homologous end joining capacity than the other cell lines. Thus, acquired chemotherapy resistant cells rely upon enhanced DNA repair in both of the main double strand break repair pathways but high levels of resistance are associated with high levels of homologous recombination activity.

To determine whether the adapted cells are reliant upon any specific pathway, we inhibited repair with specific small molecule inhibitors in the context of cell death via DNA double strand breaks induced by gamma irradiation (Figure 2E). We suggest that the inhibition of activity of these key proteins’ functions disable the related DNA repair pathway they are part of and give an estimation of that particular pathways contribution to cell survival. Each condition was standardized to vehicle only control. A cellular response to ATM inhibition would represent a general activation of double strand break response, while a response to ATR inhibition would represent a reliance upon single strand break response repair. Specific targeting of key repair proteins provides further insights. Cell death in response to DNA-PK inhibition represents a reliance upon non-homologous end joining, while cell death induced by RAD51 inhibition shows cellular reliance upon homologous recombination. Finally, PARP inhibition is a measure of reliance upon single stranded base excision repair, which upon replication fork collapse activates both homologous recombination and alternative non-homologous end joining. Cellular response, as detailed above, also provides insight into new potential targets for therapeutic intervention. Comparing cell death induced by each of DNA-PKi representing NHEJ, RAD51i representing HR and PARPi representing alt-NHEJ as a representation of the sum of the main double strand break DNA repair mechanisms at play within a cell as a percentage of the whole., we observed a switch from an equal distribution between the available repair mechanisms of alternative-non homologous end joining, non-homologous end joining and homologous recombination pathways in MDA-MB-231^SENS^ to a dominant homologous recombination dependent phenotype in MDA-MB-231^RES^ (Figure 2D). We wanted to evaluate the same response with low dose combination chemotherapy. A comparison of response between sensitive and resistant lines showed there was a maximum of 10% difference in cell death in response across all lines. The same shift towards homologous recombination was observed when DNA double strand breaks were induced by the combination of docetaxel and doxorubicin in MDA-MB-231 lines (Figure 2E). The BT549 cell line, like MDA-MB-231, also displayed significant difference in chromosome count and, consistent with this, displayed the same shift towards reliance on the homologous recombination pathway when resistant to the combination of docetaxel and doxorubicin (Figure 2F).

We next performed cell cycle analysis in response to chemotherapy to evaluate resistant cellular checkpoint stability and response to chemotherapy independent of cell death. It is interesting that little difference in cell cycle distribution is observed when comparing matched untreated sensitive and resistant lines suggesting no loss of baseline cell cycle control (Figure 2G). All sensitive cell lines responded with a G2/M phase arrest after chemotherapy treatment, while resistant cell lines displayed various levels of minor G2/M arrest up to 6–12% for MDA-MB-231, BT549 and Hs578t. The less chemoresistant MDA-MB-468 displayed +26% G2/M arrest compared to untreated (Figure 2G).

To evaluate whether the increased reliance on homologous recombination was related to higher levels of DNA damage, RAD51 function or even longer time spent in S-G2 phase where homologous recombination is active, we analyzed the resolution of DNA double strands breaks over a 24-h time course in response to 2 Gy gamma-irradiation in BT549 and MDA-MB-231 cells, respectively (Figure 2H and Supplementary Figure S4). Double strand breaks are visualized within the nucleus by gamma-H2AX foci marks. As a point for comparison, we also analysed the extent of double strand breaks induced by chemotherapy and found 1.5–2-fold more double strand breaks in BT549^SENS^ cells compared to irradiation (Supplementary Figure S5). Irradiation induces a G1-arrest and active double strand break repair is visualized and attributed to homologous recombination as RAD51 foci, while the switch to activate any non-homologous end joining is visualized with phosphorylated-DNAPK foci (Figure 2H/I) and 53BP1 foci (Supplementary Figure S4). Compared to BT549^SENS^ cells, (Figure 2H) BT549^RES^ cells (Figure 2I) had a higher RAD51 foci count per nucleus in response to irradiation, although these formed with slower kinetics than in the sensitive cells. Although the cells display slower RAD51 kinetics, BT549^RES^ cells resulted in enhanced resolution of gamma-H2Ax foci (DNA double strand breaks) at the 24-hour time point, as compared to the BT549^SENS^ cells treated with the same dose of IR (Figure 2H versus 2I). BT549^RES^ and BT549^SENS^ displayed similar kinetics of DNA-PK induction following IR treatment, however BT549^RES^ displayed very low response consistent with reduced signaling towards non-homologous end joining (Figure 2H versus 2I). This data supports our inhibitor and GFP reporter results and showed that resistant cells utilize homologous recombination, sustain the same amplitude of double strand breaks as sensitive cells but resolve these breaks more efficiently.

### Sensitization of chemoresistant cells by targeting RAD51 and homologous recombination

**Figure 3.**
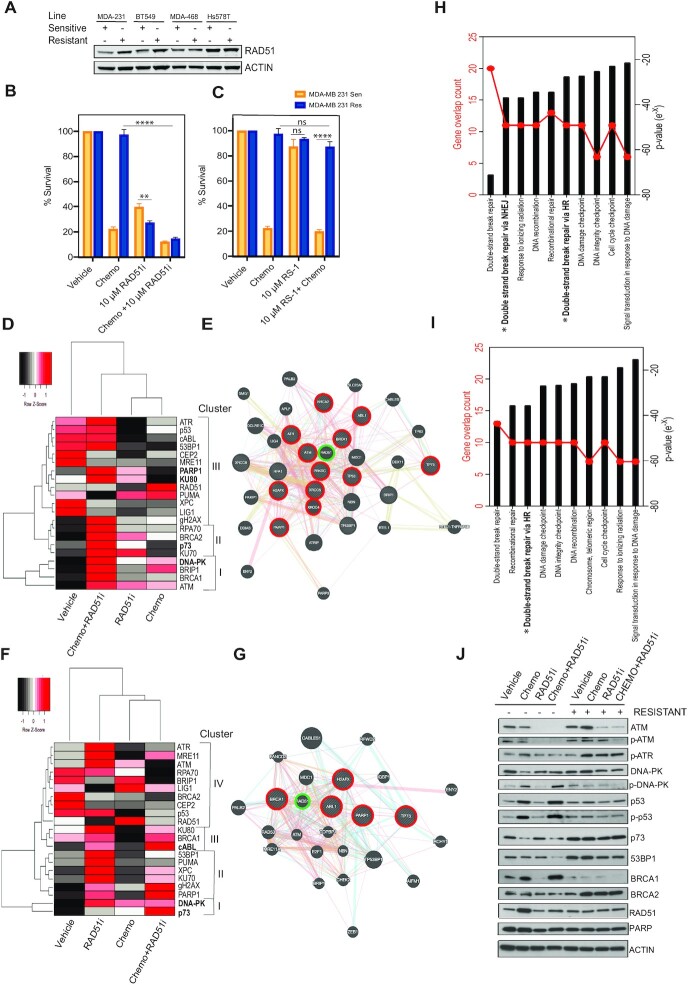
Sensitization of chemoresistant cells by targeting RAD51 and homologous recombination. (**A**) Protein expression analysis of RAD51 in all resistant and sensitive lines, actin shown as loading control. (**B**) MTT metabolism assay targeting homologous recombination with a RAD51 inhibitor (****P < 0.00001, **P < 0.001 paired student t-test, ± SEM of 3 independent experiments). (**C**) MTT metabolism assay targeting homologous recombination with a RAD51 stabilizer (****P < 0.00001 paired student t-test, ± SEM of 3 independent experiments). (**D**) Gene clustering of qPCR gene expression analysis of MDA-MB-231 sensitive cells response to chemotherapy alone and in combination (Z-score -2 to +2 per gene across 4 conditions). (**E**) Functional interaction networks distances based on physical, colocalization and coexpression values. Key gene nodes of high expression after treated with chemotherapy and RAD51i are highlighted in red. RAD51 highlighted in green. Connectors, physical interactions (pink lines) and outliers joined by pathway association (blue lines). (**F**) Gene clustering of qPCR gene expression analysis of MDA-MB-231 resistant cells response to chemotherapy alone and in combination (Z-score –2 to +2 per gene across 4 conditions). (**G**) Functional interaction networks distances based on physical (pink lines), colocalization (blue lines) and coexpression values (purple lines). Key gene nodes of high expression after treated with chemotherapy and RAD51i are highlighted in red. RAD51 highlighted in green. Connectors, physical interactions (pink lines) and outliers joined by pathway association (blue lines). (**H**) Molecular signatures Database pathway analysis using curated gene set comparing response to chemotherapy and RAD51i in MDA-MB-231 SENS cells. (**I**) Molecular signatures Database pathway analysis using curated gene set comparing response to chemotherapy and RAD51i in MDA-MB-231 RES cells. (**J**) Protein expression analysis of MDA-MB-231 resistant and sensitive cells response to chemotherapy alone and in combination.

Having established an enhanced homologous recombination repair phenotype in chemoresistant cell lines, we first analysed the expression levels of RAD51 across all cell lines. Moderate RAD51 expression increases were observed in the lines that achieved high levels of resistance, MDA-MB-231^RES^ and BT549^RES^ (Figure 3A). Next we wondered if targeting of the key repair protein RAD51 could sensitize these cells. Utilizing a validated small molecule inhibitor of RAD51 (22), MDA-MB-231^RES^ were actually significantly more sensitive to the inhibitor than MDA-MB-231^SENS^ cells (Figure 3B). The combination of chemotherapy and RAD51 inhibition achieved resensitization and equivalent levels of cell death independent of chemoresistance status (Figure 3B). Conversely, stabilization of RAD51 with a small molecule (RS-1) (23) congruently enhanced chemotherapy resistance in MDA-MB-231^RES^ (Figure 3C). We observed the same results in the BT549 matched lines (Supplementary Figure S6). Therefore, RAD51 function demonstrated a direct role of homologous recombination in chemoresistance.

RAD51 alone is a useful marker for HR deficiency/proficiency however it is upregulated in response to any DNA damage whether the source is endogenous or exogenous and thus limiting as a predictor of response alone. Therefore, we suggest the ability to utilize gene expression profiling in response to targeting RAD51 has the potential to track patient response and relapse guiding clinical choices. The targeting of RAD51 with chemotherapy induced cell death irrespective of chemoresistance. This provides the capability to successfully target the heterogeneity often observed in TNBC. However, we find that the functionally available repair pathways in sensitive and resistant lines are very different. We evaluated the gene expression profile as a readout for DNA damage response to the combination of chemotherapy and RAD51i (Figure 3B). Utilizing a targeted array of 21 DNA repair related genes and cluster analysis per individual gene in MDA-MB-231^SENS^ cells we revealed 15/21 of the genes were most highly induced by chemotherapy and RAD51i (Figure 3D). The Euclidean distance arranged these genes into three main clusters, each of which were derived of both key non-homologous end joining and homologous recombination pathways, cluster I-BRCA1 and DNA-PK, cluster II-KU70 and BRCA2 and cluster III PARP1 and KU80. This was representative of an even distribution of DDR pathways utilized and similar to what was observed in our small molecule inhibition studies in MDA-MB-231^SENS^ cells (Figure 2F). The induction of gene expression is a representation of individual gene response however we were curious about the functional response. Therefore, we performed functional interaction network analysis and displayed distances based on physical, colocalization and co-expression values. The most highly expressed genes circled in red were tightly associated by physical interactions (pink lines) and outliers joined by pathway association (blue lines) (Figure 3E).

Analysis of MDA-MB-231^RES^ cells, revealed only 5/21 of the genes were most highly induced by chemotherapy and RAD51i (Figure 3F). These genes were functionally associated with inhibition of non-homologous end joining, cluster I-p73, cluster II-PARP1, cluster III-cABL1 and no highly expressed genes in cluster IV (Figure 3F). Functional interaction network analysis revealed that RAD51 was again centrally located in the network and closely associated with cABL1 and BRCA1 (Figure 3G). Notably RAD51 was also induced in response to chemotherapy alone in both cell lines and as it is centrally located in each network it is a rationale target in TNBC tumours that often display chemoresistance heterogeneity.

Gene ontology molecular functions analysis of the 21 genes revealed that the MDA-MB-231^SENS^ continued to rely upon both homologous recombination and non-homologus end joining as two of the top 10 pathways while MDA-MB-231^RES^ relied upon only homologous recombination (Figure 3H/I). Supporting protein analysis of MDA-MB-231^RES^ revealed that DNA-PK did not display the classical phosphorylation in response to chemotherapy as seen in MDA-MB-231^SENS^, although total DNA-PK protein expression was sustained (Figure 3J). This suggested non-functional or ablated non-homologous end joining activity. We also observed associated increases in 53BP1 protein expression in MDA-MB-231^RES^ across all treatment conditions supporting activation of DNA-PK and non-homologous end joining activity (Figure 3J). RAD51 protein expression was not enhanced and there was an associated loss of BRCA1 expression, suggesting suppression of homologous recombination. However, we also observed a compensation by enhanced BRCA2 expression in MDA-MB-231^RES^ cells. In addition, we observed an induction of p73 with associated repression of p53 activation, providing a switch in DNA damage sensing to p73.

## DISCUSSION

The TNBC cell lines utilized in this study span several molecular TNBC subtypes. MDA-MB-231 and HS578t have molecular features of the mesenchymal stem-like (MSL) subtype, BT549 is mesenchymal (M), and MDA-MB-468 is categorised as basal-like 1 (BL1) (24). Interestingly BL1 tumours were characterised by high genomic instability which may be why we were unable to induce further chemoresistance (25). By choosing TNBC cell lines with different molecular characteristics we aimed to investigate whether changes in DNA repair function were a universal sign of intrinsic chemoresistance. Our previous work supports the hypothesis that high RAD51 expression and homologous recombination reliance is a late stage event supporting patient relapse and metastasis (7). Our current findings show early reliance upon non-homologous end joining and DNA-PK activity under genome instability conditions resulted in acquisition of chemoresistance phenotypes and changes in genetics including loss of chromosome count. In support of genomic instability and DDR driving early drug adaption, Hansen *et al.* identified major genomic variations midway through docetaxel adaption (26) and Tsou *et al.* the attenuation of DNA repair proteins BRCA1/2 and wild type p53 early in adaption to doxorubicin (27).

**Figure 4.**
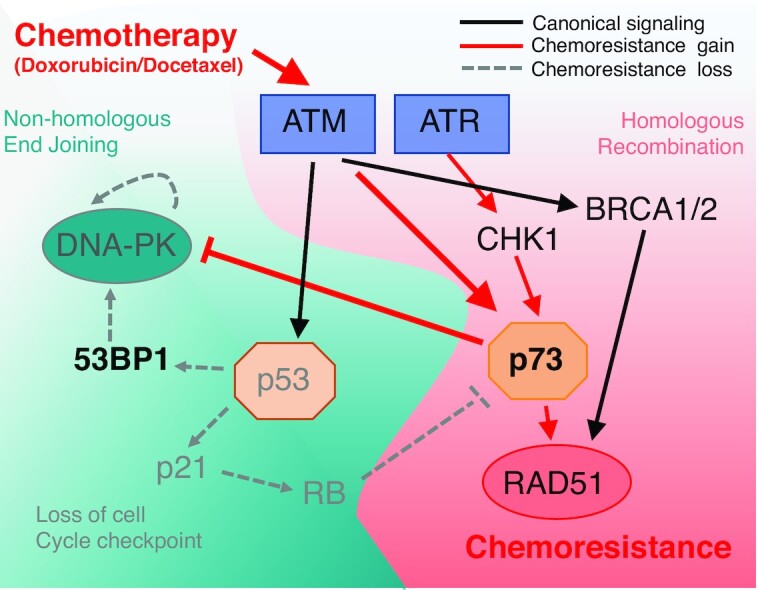
Pathway analysis under treatment conditions and resistance associated with induction of chemoresistance. Schematic diagram of chemoresistance signaling based on functional gene analysis and gene expression profiling observed in TNBC adapted cell lines.

We suspected from hierarchical clustering that loss of mutant p53 and DNA-PK dysfunction are early events followed by reduced 53BP1 expression in selected clonal expansion rather than general pathway regulation (Figure 4). TNBC patient tumours and derived cell lines each harbour high levels of replication stress. Replication stress, is defined by a dependence upon ATR activation due to extended single stranded DNA at the replication fork (28). Therefore baseline levels of aberrant DNA repair is already high and associated chromosome instability can be tolerated promoting diversification of subclones (29). Our TNBC sensitive cell lines displayed instability with baseline ATR dependence and therefore were primed for genetic loss. In a recent melanoma study, Kwong *et al.* showed under strong selective pressure, genetically stable tumours (diploid) acquired resistance via mutation and activation of oncogenic pathways, whereas genomically unstable tumours acquired resistance via broad whole chromosome aneuploidy (30). This mechanism was also reflected in TNBC patients with poor response to chemotherapy associated with higher levels of aneuploidy prior to treatment (31). This suggests clonal diversity is derived from pressure on the DDR and aberrant DNA repair preceding multidrug resistance phenotypes.

Almost all TNBC harbor TP53 mutations, with 82% of patients displaying somatic alterations (6). Most TP53 mutations confer resistance to drugs that directly target DNA or DNA synthesis (32). In our study, the lines that achieved the highest levels of resistance, MDA-MB-231 and BT549, have R280K and R249S p53 gain-of-function missense mutations, respectively. These mutations reduce DNA binding and infers reduced activation of cell death pathways (33,34). Cancer cells that harbor mutation of p53 modulate induction of apoptosis through p73 (35). p73 is most commonly thought to act as a tumour suppressor via induction of cell death pathways under oncogenic stress. We observed a loss of function in mutant p53 after drug adaptation, however with no associated induction of apoptosis via p73 (36). This is likely due to the exclusive expression of the ΔNp73 isoform that acts as a dominant-negative inhibitor. ΔNp73 competes with p53, TAp63 and TAp73 for promoter binding and inhibits the activation of target genes, thereby blocking apoptosis (37). Indeed, we did not see induction of the classical apoptosis target gene PUMA in any of the cell lines we tested. In addition to evading apoptosis, we suggest that ΔNp73 contributed to the switch to dependence on homologous recombination. In contrast to TAp73, ΔNp73 binds 53BP1 inhibiting function, therefore depleting 53BP1 foci recruitment at DSBs and repressing non-homologous end joining (38). Therefore, in contrast to serving as a tumour suppressor, we and others suggest that ΔNp73 acts as an oncogene (39) notably during emergence of chemoresistance and DNA damage response crisis switch.

DNA-PK has been shown to be important for telomere maintenance and therefore we suggest inactivation further supports genetic loss (40). However, it is also plausible that in conditions of constant genotoxic stress, overactive NHEJ repair could have been a survival hindrance due to constant loss of chromosomes and/or parts of chromosomes increasing the reliance on HR as a more accurate DNA repair mechanism to preserve genetic information. In addition, we saw changes in proliferation of chemoresistant cells in comparison to chemosensitive cells. The differences were most profound in the MDA-MB-231 pair, where resistant cells grew approximately 2.5 times more slowly than sensitive. Gene expression analysis of MSL tumours showed that they retained *RB1* while displaying significantly lower *CDK4* and *CDK6* expression levels, which means they have a pre-set level for reduced cell cycle progression (41). We suspect that under higher levels of genotoxic stress cells have higher rates of cell cycle checkpoint activation which ultimately impedes cell cycle progression and provides a temporal prerequisite for DNA repair by HR. We observed a similar functional shift towards HR in mesenchymal chemoresistant BT549 cells, however in contrast to MSL subtype the mesenchymal BT549^RES^ increased their cell cycle rate. The mesenchymal TNBC subtype displays overexpression of the *MYC* oncogene, not observed in the MSL subtype and likely supports cell cycle progression (41).

Targeting the DDR to resensitize chemoresistant cancers is yet to be fully realized. One of the few clinical examples demonstrated a significant increase in objective response activity in platinum-resistant ovarian cancer patients when a WEE1 inhibitor was added (42). The targeting of DNA-PK has yet to be tested in breast cancer. Some efficacy has been shown against haematological malignancies and activity in vitro against MDA-MB-231, however we would be concerned about promoting the resistant profile we defined. In support of this, we were able to recapitulate the chemoresistance profile by targeting DNA-PK and creating a resistant sub-population (Supplementary Figure S7). We suggest the downstream homologous recombination effector, RAD51, as a rational clinical target to re-sensitize to chemotherapy in a contextual synthetic lethality response (43). Several strategies for targeting RAD51 are under development including small molecule inhibitors and antibody fragments (44,45). Currently an oral RAD51 inhibitor CYT-0851 is being tested in a Phase 1/2 study against relapsed/refractory B-cell malignancies and advanced solid tumours including breast cancer (NCT03997968). We eagerly await the results and suggest its use for refractory TNBC.

## DECLARATIONS

### Consent for publication

All authors have read the manuscript and agree to submission.

## DATA AVAILABILITY

All authors agree to open access to data and materials. The datasets generated for Figure 1A–D are available in the NCBI GEO ‘Genomic predictor of response and survival following neoadjuvant taxane-anthracycline chemotherapy in breast cancer, https://www.ncbi.nlm.nih.gov/geo/query/acc.cgi?acc=gse25066.

## Supplementary Material

zcab022_Supplemental_Files
