# Supplementary material for: Genome instability and pressure on non-homologous end joining drives chemotherapy resistance via a DNA repair crisis switch in triple negative breast cancer
Source: NAR Cancer. 2021 Jun 15;3(2):zcab022. doi: 10.1093/narcan/zcab022 (PMC8210242; doi:10.1093/narcan/zcab022)
Supplement: zcab022_Supplemental_Files [file zcab022_supplemental_files.zip › Wiegmans Ward Supplementary files R2.docx]

**Supplementary Information.**

**Metaphase spreads**

Exponentially growing cells were treated with 1 μg/ml of colcemid for 3-4 hours. Cells were collected and incubated in hypotonic solution (0.56% KCl) for 6 minutes, fixed in methanol: glacial acetic acid (3:1), spotted onto glass slides and air-dried. To visualize metaphase spreads the chromosomes were stained with DAPI (1:1000 in PBS) and viewed under a Zeiss AxioScop2 fluorescent microscope. Chromosomes were counted for 20 metaphase spreads.


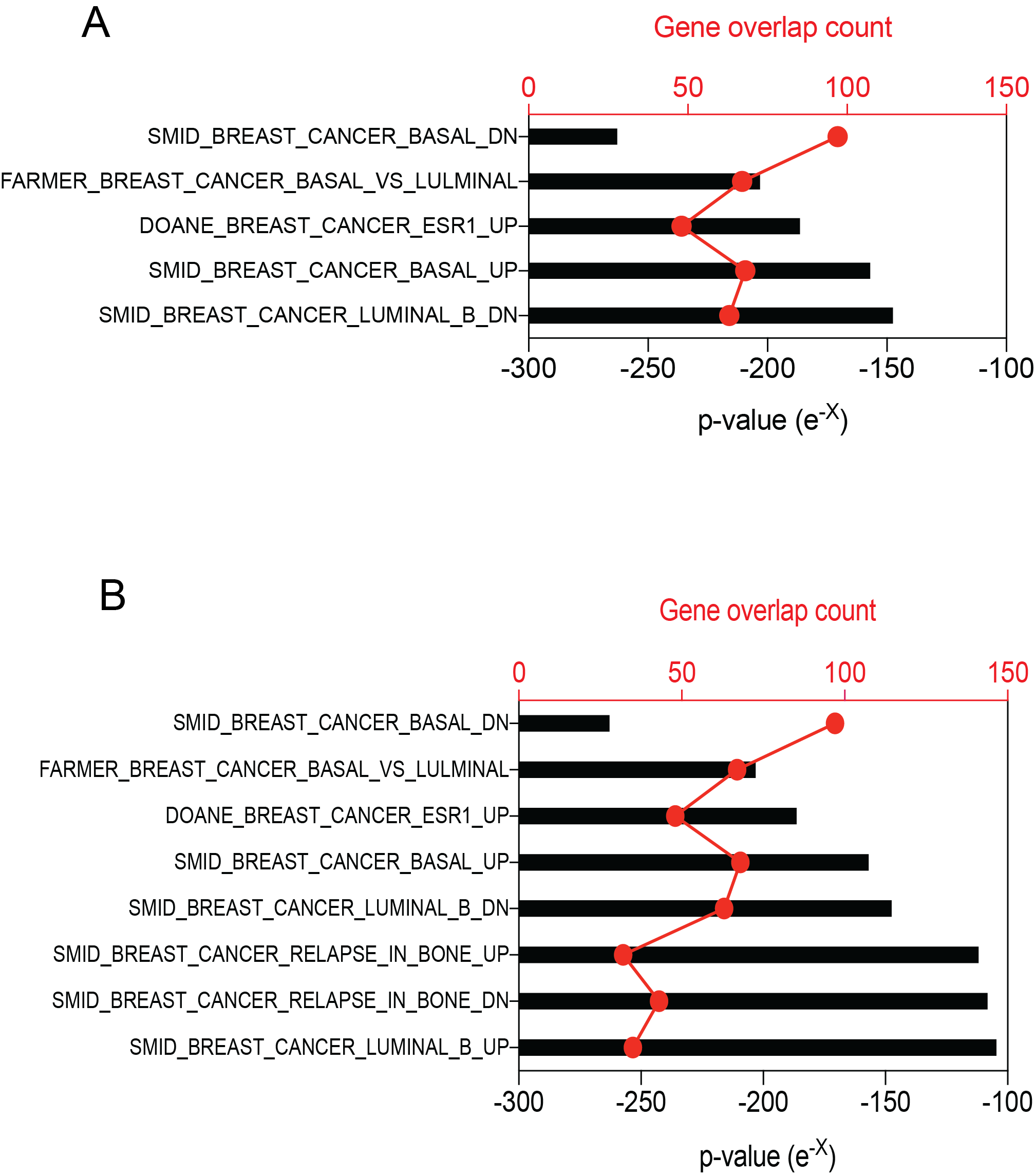


**Supplementary Figure 1.** Molecular signatures Database curated gene set comparing TNBC to Luminal/HER2+ subtypes for gene expression changes in molecular functions ranked based on gene numbers and statistical significance following neoadjuvant taxane-anthracycline chemotherapy (GSE25066) using;

**A** Molecular Signature database curated top 50 gene expression datasets based on p-value and false discovery rates (Mann-Whitney *U* test p-values as shown).

**B** Molecular Signature database top 50 breast cancer gene expression datasets.


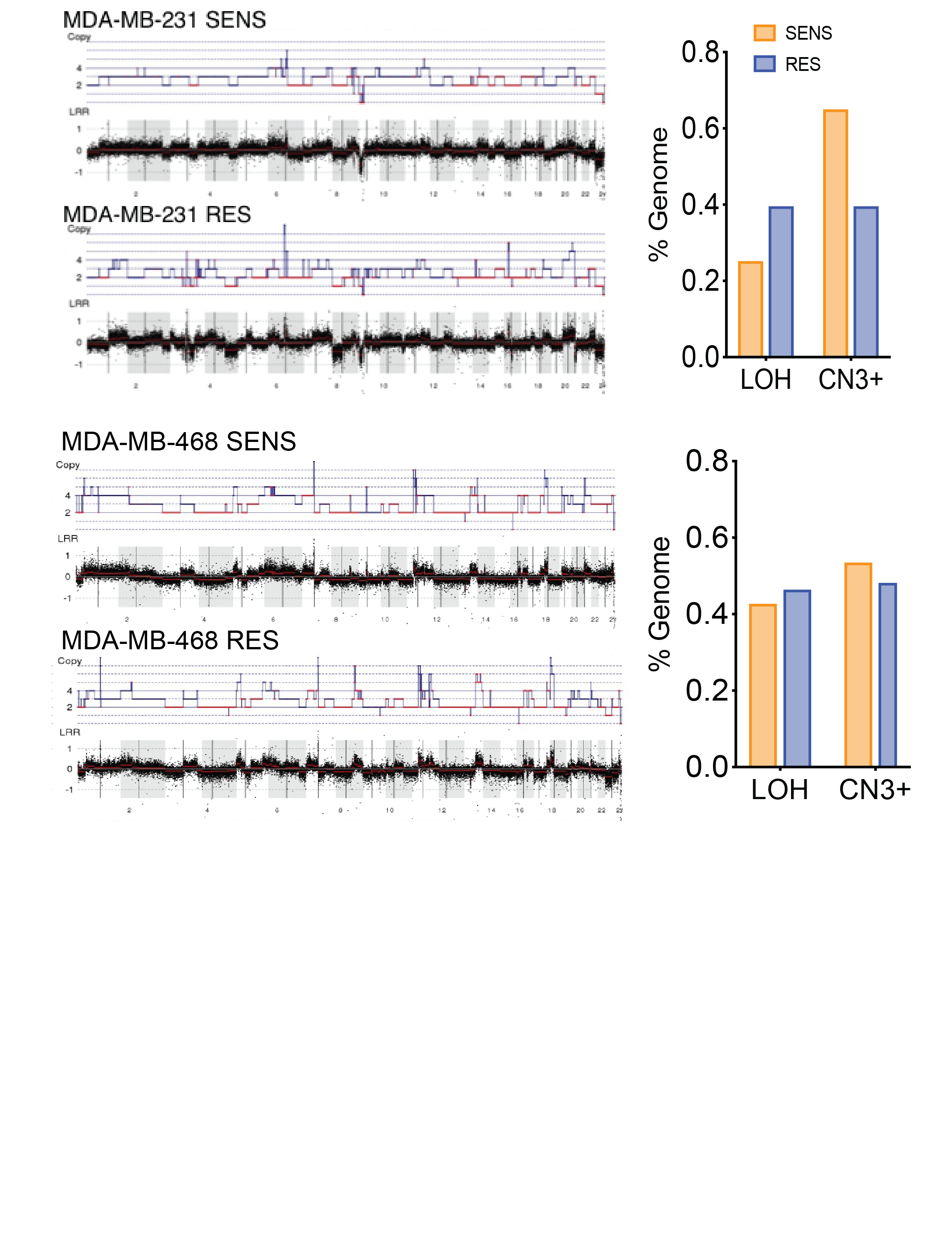


**Supplementary Figure 2.** Copy number array results displaying gains in copy number in allele A compared between sensitive and resistant cell lines of MDA-MB-231 and MDA-MB-468. LOH: Loss of Heterozygosity, CN3+: Genes that display an increase in copy number of 3 or more copies.

**Supplementary Figure 3.** Correlations between the AW signature from chemotherapy treated TNBC patients and a measure for:

**A** ploidy status binned as "diploid", below diploid ("aneup_lo"), above diploid ("aneup_high") and as well all aneuploid samples (sum of latter two bins),

**B** a chromosome instability signature (CIN70),

**C** ploidy status, and

**D** chromosome arm-level somatic copy number alteration burden (cal_scna_burden), Data are from TCGA basal breast cancers. *R* and *p* values: Spearman's rank correlations. Samples sizes, n, are also shown.

**
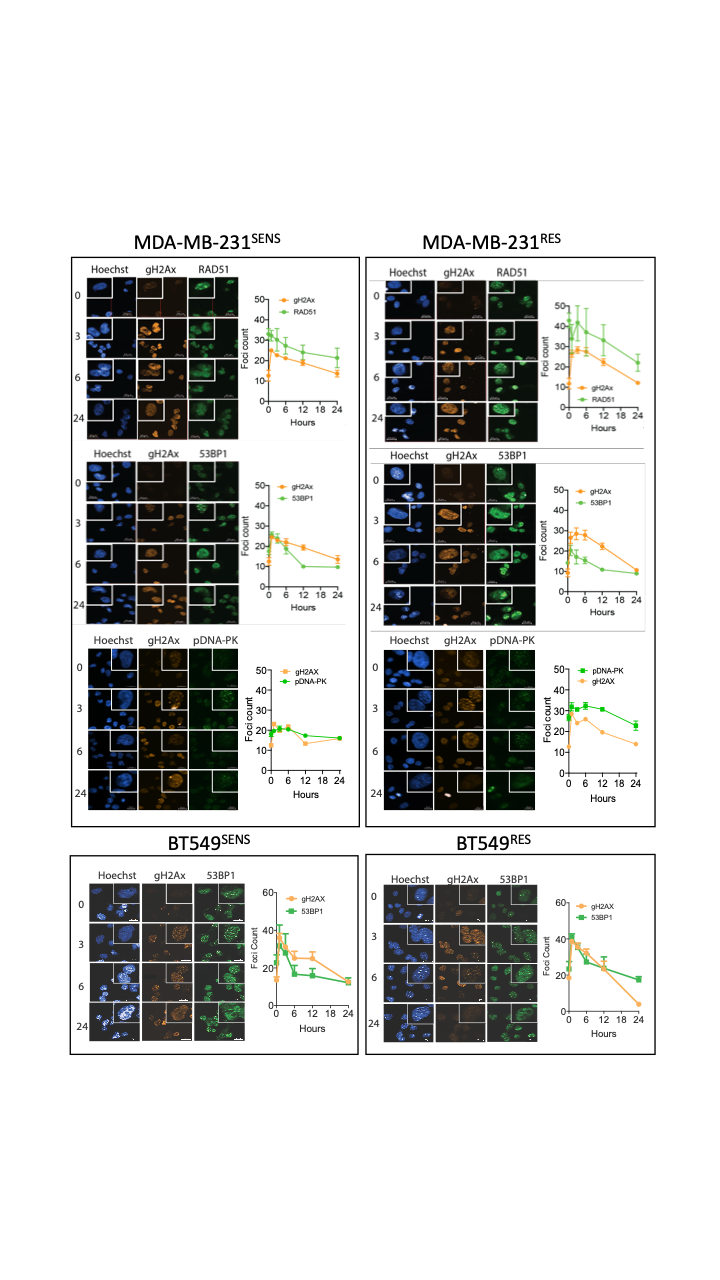
**

**Supplementary Figure 4.** Immunofluorescent analysis of the rate of double strand break repair marked by comparison of gammaH2AX foci marked breaks, homologous recombination RAD51 foci, non-homologous end joining pDNA-PK foci and DNA repair switch 53BP1 foci markers in MDA-MB231 cells and 53BP1 foci in BT549 cells.

**Supplementary Figure 5.** Immunofluorescent analysis of the rate of double strand break repair marked by comparison of gamma-H2AX foci marked breaks in response to chemotherapy in BT549 cells. Samples were read at 30min, 1hr, 6hrs, 24hrs and 48hrs after treatment with the combination of 30nM doxorubicin and 0.3nM docetaxel. Plot is average of 3 independent experiments +/- SEM.

**Supplementary Figure 6. Sensitization of chemoresistant cells by targeting RAD51 and homologous recombination.**

**A** Metabolism assay targeting homologous recombination with a RAD51 inhibitor (*****p<*0.00001, ***p<*0.001 paired student t-test, +/- SEM of 3 independent experiments).

**B** Metabolism assay targeting homologous recombination with a RAD51 stabilizer (*****p<*0.00001 paired student t-test, +/- SEM of 3 independent experiments).

**Supplementary Figure 7.** **Resistance to DNA-PK inhibition recapitulates the chemoresistance phenotype.**

**A** MDA-MB-231 DR-GFP SENS cells were treated with escalating doses of DNA-PKi over 3 weeks to establish cells that have adapted to NHEJ inhibition. This inhibition resulted in significant resistance to high dose chemotherapy (100nM doxorubicin and 1nM), two tailed student t-test.

**B** DNA repair GFP reporter analysis of HR and NHEJ of adapted cell line compared to MDA-MB-231 SENS.

**Supplementary Table 1. Gene list of differential gene expression in TNBC versus the other breast cancer subtypes.**

Gene set analysis of clinical trial data GSE25066 resulted in 88 genes induced while 130 genes were repressed.

| **Gene** | **Pathway** | **Genomic location** | **Copy number** | | | |
| --- | --- | --- | --- | --- | --- | --- |
|  |  |  | **231 Sen** | **231 Res** | **468 Sen** | **468 Res** |
| *p53* | p53 family | chr17: 7,661,779 -7,687,550 | 3 | 2 | 2 | 2 |
| *p63* | p53 family | chr3: 189,631,389-189,897,276 | 3 | 2 | 2 | 2 |
| *p73* | p53 family | chr1: 3,652,516-3,736,20 | 2 | 2 | - | - |
| *MDM2* | p53 regulator | chr12: 68,808,177-68,850,686 | 2 | 2 | 2 | 2 |
| *ATR* | ATR-CHK1 pathway | chr3: 142,449,007-142,578,733 | 2 | 2 | 3 | 3 |
| *CHK1* | ATR-CHK1 pathway | chr11: 125,625,665-125,676,255 | 4 | 3 | 4 | 3 |
| *ATM* | ATM-CHK2 pathway | chr11: 108,222,484-108,369,102 | 4 | 4 | 4 | 4 |
| *CHK2* | ATM-CHK2 pathway | chr22: 28,687,743 -28,742,422 | 2 | 2 | 3 | 3 |
| *CDC25A* | Checkpoint | chr3: 48,157,146-48,188,402 | 3 | 2 | 2 | 2 |
| *WEE1* | Checkpoint | chr11: 9,595,228-9,615,004 | 3 | 3 | 2 | 2 |
| *p16* | Senescence | chr9: 21,967,753-21,995,301 | 0 | 0 | 2 | 2 |
| *p19* | Senescence | chr19: 10,566,462-10,569,059 | 2 | 2 | 5 | 3 |
| *FAS* | Apoptosis | chr10: 88,990,531-89,017,059 | 3 | 3 | 3 | 3 |
| *NOXA* | Apoptosis | chr18: 59,899,948-59,904,306 | 2 | 3 | 2 | 2 |
| *PUMA* | Apoptosis | chr19: 47,220,822-47,232,766 | 3 | 2 | 3 | 3 |
| *BAX* | Apoptosis | chr19: 48,954,815-48,961,798 | 3 | 2 | 3 | 3 |
| *BIK* | Apoptosis | chr22: 43,110,750-43,129,712 | 2 | 2 | 3 | 2 |
| *BCL2* | Apoptosis | chr18: 63,123,346-63,320,128 | 2 | 3 | 2 | 2 |
| *MLH2* | MMR | chr2: 47,402,969-47,663,146 | 3 | 2 | 4 | 4 |
| *MLH1* | MMR | chr3: 36,993,350-37,050,846 | 3 | 2 | 2 | 2 |
| *XPC* | NER | chr3: 14,145,145-14,178,672 | 3 | 3 | 2 | 2 |
| *XPG* | NER | chr13: 102,807,146-102,876,001 | 2 | 2 | 4 | 5 |
| *XPF* | NER | chr19: 45,407,333-45,478,828 | 3 | 2 | 4 | 4 |
| *XRCC1* | BER | chr19: 43,543,040-43,580,473 | 3 | 2 | 4 | 3 |
| *PARP1* | BER, alt-NHEJ | chr1: 226,360,691-226,408,093 | 3 | 3 | 4 | 3 |
| *DNA-PK* | NHEJ | chr8: 47,773,108-47,960,183 | 2 | 1 | 2 | 2 |
| *KU70* | NHEJ | chr22: 41,621,119-41,664,048 | 2 | 2 | 3 | 2 |
| *KU80* | NHEJ | chr2: 216,107,464-216,206,303 | 3 | 3 | 3 | 3 |
| *53BP1* | NHEJ | chr5: 43,403,061-43,510,728 | 3 | 2 | 3 | 3 |
| *MRE11* | HR | chr11:94,415,578-94,493,908 | 4 | 4 | 2 | 4 |
| *BRCA2* | HR | chr13: 32,315,086-32,400,266 | 2 | 2 | 2 | 2 |
| *RAD51* | HR | chr15: 40,694,774-40,732,340 | 2 | 2 | 2 | 2 |
| *RPA1* | HR | chr17: 1,829,702-43,170,245 | 3 | 2 | 2 | 2 |
| *BRCA1* | HR | chr17: 43,044,295-43,170,245 | 3 | 2 | 2 | 2 |

**Supplementary Table 2. DNA repair associated gene copy number analysis.**
